# Wearable Neck Surface Accelerometers for Occupational Vocal Health Monitoring: Instrument and Analysis Validation Study

**DOI:** 10.2196/39789

**Published:** 2022-08-05

**Authors:** Zhengdong Lei, Lisa Martignetti, Chelsea Ridgway, Simon Peacock, Jon T Sakata, Nicole Y K Li-Jessen

**Affiliations:** 1 School of Communication Sciences and Disorders McGill University Montreal, QC Canada; 2 School of Medicine University of Montreal Quebec, QC Canada; 3 The Alliance of Canadian Cinema, Television and Radio Artists – Montreal Montreal, QC Canada; 4 Department of Biology McGill University Montreal, QC Canada; 5 The Centre for Research on Brain, Language and Music McGill University Montreal, QC Canada; 6 Department of Biomedical Engineering McGill University Montreal, QC Canada; 7 Department of Otolaryngology - Head and Neck Surgery McGill University Montreal, QC Canada; 8 Research Institute of McGill University Health Center Montreal, QC Canada

**Keywords:** mechano-acoustic sensing, voice monitoring, wearable device, neck surface accelerometer

## Abstract

**Background:**

Neck surface accelerometer (NSA) wearable devices have been developed for voice and upper airway health monitoring. As opposed to acoustic sounds, NSA senses mechanical vibrations propagated from the vocal tract to neck skin, which are indicative of a person’s voice and airway conditions. NSA signals do not carry identifiable speech information and a speaker’s privacy is thus protected, which is important and necessary for continuous wearable monitoring. Our device was already tested for its durable endurance and signal processing algorithms in controlled laboratory conditions.

**Objective:**

This study aims to further evaluate both instrument and analysis validity in a group of occupational vocal users, namely, voice actors, who use their voices extensively at work in an ecologically valid setting.

**Methods:**

A total of 16 professional voice actors (age range 21-50 years; 11 females and 5 males) participated in this study. All participants were mounted with an NSA on their sternal notches during the voice acting and voice assessment sessions. The voice acting session was 4-hour long, directed by a voice director in a professional sound studio. Voice assessment sessions were conducted before, during, and 48 hours after the acting session. The assessment included phonation tasks of passage reading, sustained vowels, maximum vowel phonation, and pitch glides. Clinical acoustic metrics (eg, fundamental frequency, cepstral measures) and a vocal dose measure (ie, accumulated distance dose from acting) were computed from NSA signals. A commonly used online questionnaire (Self-Administered Voice Rating questionnaire) was also implemented to track participants’ perception of vocal fatigue.

**Results:**

The NSA wearables stayed in place for all participants despite active body movements during the acting. The ensued body noise did not interfere with the NSA signal quality. All planned acoustic metrics were successfully derived from NSA signals and their numerical values were comparable with literature data. For a 4-hour long voice acting, the averaged distance dose was about 8354 m with no gender differences. Participants perceived vocal fatigue as early as 2 hours after the start of voice acting, with recovery 24-48 hours after the acting session. Among all acoustic metrics across phonation tasks, cepstral peak prominence and spectral tilt from the passage reading most closely mirrored trends in perceived fatigue.

**Conclusions:**

The ecological validity of an in-house NSA wearable was vetted in a workplace setting. One key application of this wearable is to prompt occupational voice users when their vocal safety limits are reached for duly protection. Signal processing algorithms can thus be further developed for near real-time estimation of clinically relevant metrics, such as accumulated distance dose, cepstral peak prominence, and spectral tilt. This functionality will enable continuous self-awareness of vocal behavior and protection of vocal safety in occupational voice users.

## Introduction

### Background

Neck surface accelerometers (NSAs), a type of mechano-acoustic sensor, have been adopted as mobile health (mHealth) wearables for voice and upper airway health monitoring [1-5]. The vocal folds, which are housed in the larynx, oscillate at high frequencies (>100 Hz) when we speak or sing. The generated acoustic waves travel along the vocal tract, which acts as a resonator to shape the sound into audible speech. Concurrently, these acoustic waves propagate laterally to the tracheal wall and the neck skin surface. NSAs are used to convert these mechanical accelerations into electrical signals for digital devices, which can be applied to monitor a person’s vocal activity and health.

Compared with other mHealth wearables embedded with acoustic microphones, NSA-based wearables have advantages of protecting speaker’s privacy and increasing signal quality for remote, continuous voice monitoring. For instance, the neck tissue acts as a low-pass filter by nature and restricts the signal bandwidth to 1.5 kHz at maximum [4]. As most recognizable phonetic features (eg, vowel formants) are within the high-frequency range (around 6-8 kHz), identifiable speech information is already filtered by the neck tissue and barely captured by NSAs [6]. Furthermore, NSAs possess anti-interference ability against background noise because they are only sensitive to contact vibration but not to air-borne acoustic waves.

From the clinical perspective, an individual’s voice condition is evaluated through an array of acoustic and aerodynamic metrics such as fundamental frequency (*f*_0_), cepstral peak prominence (CPP), sound pressure level (SPL), subglottal pressure as well as the difference between the first and second harmonic magnitudes (H1 – H2). These clinical metrics are typically obtained from conventional clinical instruments such as Computerized Speech Lab, electroglottography, and Rothenberg mask systems. These instruments are, however, large and expensive, which are not suitable for mHealth apps. Several research groups, including our team, have thus developed compact and lightweight NSA wearables to collect voice-related metric data continuously without causing users’ discomfort or interruptions to their daily activity [3,4,7-11].

Voice-related metrics obtained from NSA devices were not found to differ from those obtained with conventional instruments [12-14]. For instance, NSA-derived and microphone-derived jitter and CPP values were relatively comparable across vowels in both normal and deviated voices (both *r*>0.78) [12]. Estimation error of SPL from NSA signals of voiced speech was less than 2.8 dB [13]. To estimate aerodynamic features of voice sounds, an impedance-based inverse filtering model was applied to derive glottal volume velocity from NSA signals [15]. NSA-derived and airflow-derived H1 – H2 values were found fairly comparable (*r*=0.72) [16]. Similarly, moderate correlation (*R*^2^=0.63) was reported between the NSA root-mean-square amplitude and intraoral pressure in vocally healthy speakers across vowels [17].

Growing evidence further supports the robustness of NSA signals in discerning normal versus deviated vocal health conditions. For example, one study collected NSA-derived acoustic metrics from a group of female patients with hyperfunctional voice disorders and their matched controls for over a week [18]. The patient group displayed overall higher SPL values and less H1 – H2 variability than matched controls [18]. By applying machine learning techniques, our group showed that distinctive voice types (normal, breathy, and pressed voice) could be classified from NSA signals with more than 80% accuracy [4]. That said, in reviewing published studies using conventional air microphones, inconsistent calculated values of acoustic voice metrics were reported between sustained vowels and continuous speech [19]. Although sustained vowel tasks were more common in clinical voice assessment, continuous speech tasks are more ecologically valid to represent an individual’s natural speaking voice. A thorough evaluation of NSA-derived acoustic metrics across phonation tasks is thus imperative as part of instrument validation.

One target clinical population for voice monitoring wearables includes those who use their voices heavily in the workplace. Voice actors, singers, and teachers are examples of occupational voice users who tend to develop vocal fatigue and disorders [20-23]. A key functionality of NSA wearables is to provide real-time alerts when a user’s vocal safety limit is reached at workplace. That way, the user can take immediate action rather than unknowingly surpass the threshold for safe voice use, which would result in chronic vocal fatigue and irreversible vocal injury. Vocal dose metrics are available to estimate the amount of voice use by quantifying the distance that vocal fold travels during phonation. Several vocal dose metrics such as distance dose (Dd), cycle dose, and time dose were successfully derived from NSA signals by our group and others [11,24-27]. However, inconclusive literature suggested that these metrics could be gender dependent, which may implicate the need for creating gender-specific vocal safety limits [28]. An investigation on the quantitative relationship between vocal doses and NSA-derived acoustic metrics in both females and males is thus pivotal to validate this critical question.

### Research Objectives and Hypotheses

This study represents our ongoing work to develop and validate an in-house NSA wearable system for voice and upper airway health monitoring. Our device has already been tested in controlled laboratory settings [4,11]. As one major application of this device is to monitor voice use at workplace, the next logical step would be to test whether the device could endure one such ecologically valid condition. This study thus aimed to test the instrument validity of our NSA wearables in a group of occupational voice users, namely, voice actors, during their voice acting routines in a professional sound studio.

Briefly, all participants were subject to a voice acting session in an ecologically valid setting plus 2 follow-up sessions of voice assessments. Vocal doses and acoustic metrics were collected with our in-house NSA wearables and self-perceived vocal fatigue was assessed by an online questionnaire. NSA acoustic metrics were extracted from both sustained vowels and passage reading tasks. Furthermore, certain voice actors have a routine of practicing vocal warm-up exercise as part of their acting. Participants were thus randomized to either a warm-up group or no warm-up group before their acting session to protect the ecological validity while minimizing potential confounding effects from an individual’s warm-up history.

We hypothesized that NSA-derived acoustic metrics and self-perceived vocal fatigue ratings would show similar trends indicative of vocal fatigue and recovery. We also hypothesized that the acoustic metrics derived from passage readings would be comparable to those derived from sustained vowels. We further hypothesized that distance dose and NSA-derived acoustic metrics would be comparable between female and male participants in this study.

## Methods

### Hardware

**Figure 1:**
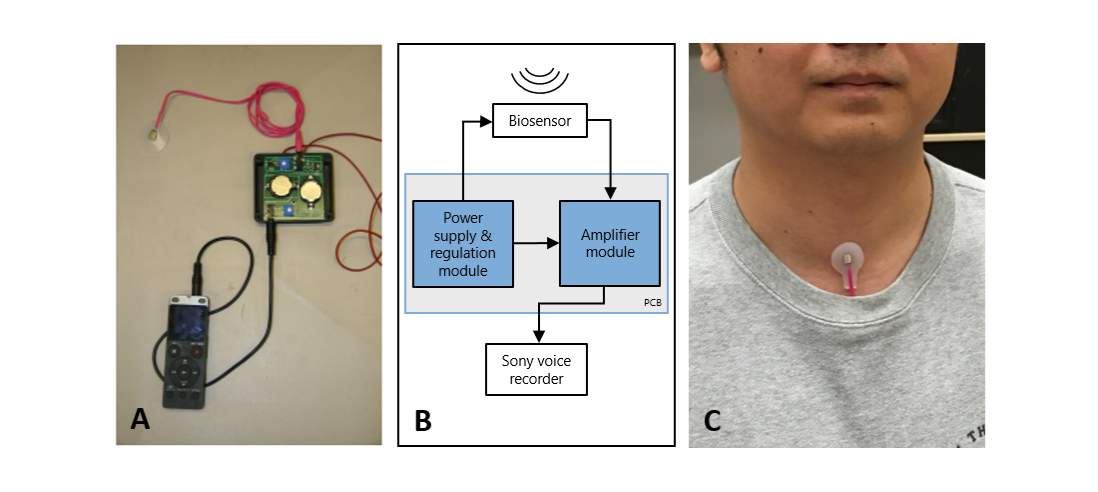
The NSA Wearable Device. (A) Hardware instrument, and (B) Schematic design. Adapted from “Figure 1. The physical prototype and schematic of the NSA”, by Lei et al, 2019 [4] and licensed under CC BY 4.0. PCB: printed circuit board.

Our in-house NSA system consisted of (1) an accelerometer (BU-27135; Knowles Inc.) set into a circular silicon pad with a diameter of 28 mm, thickness of 1.2 mm, and weight less than 20 g; and (2) a peripheral circuit containing 1 power supply module and 1 amplifier module on a printed circuit board (Figure 1). Four lithium coin batteries (CR2032; Panasonic Inc.) with a nominal voltage of 3 V and capacity of 225 mA hour were used as a power source. The peripheral circuit board was interfaced with the accelerometer using a 3.5-mm stereo audio cable. A Sony voice recorder (ICD-UX565F; Sony Inc.) was used as a data logger to save the NSA data in .wav audio format and transferred to a computer for signal processing and analysis. The total cost of each device was about CAD $100 (US $77). All NSA recordings were made using a linear pulse code modulation encoding mode with a 44.1-kHz sampling rate. A signal-to-noise ratio of 45 dB was achieved using the recorder’s multiple modes for background noise suppression. Further details and verification tests of the NSA system were reported in our previous publications [4,5,11,29].

### Participants

Participants were recruited via the Alliance of Canadian Cinema, Television and Radio Artists (ACTRA) (Montreal Chapter) network. A total of 16 professional voice actors aged 21-50 years consented to participate in the experiment. Participants were randomly assigned to either a *no warm-up* group (n=4 for both females and males) or a *warm-up* group (females: n=7; males: n=1). All participants had basic voice acting experience defined as (1) having participated in at least one voice acting workshop organized by the ACTRA; or (2) having been contracted, on at least one occasion, to complete paid voice work on a project. All reported normal hearing bilaterally. Individuals with a smoking habit (>1 cigarette per day within the last year or any smoking habit within the last 2 months), current history of chronic (ie, lasting >2 weeks) voice problems, or current use of medications that are considered to possibly affect an individual’s voice (ie, diuretics, decongestants) were excluded from the study.

### Experimental Design and Data Acquisition

#### Overview

**Figure 2:**
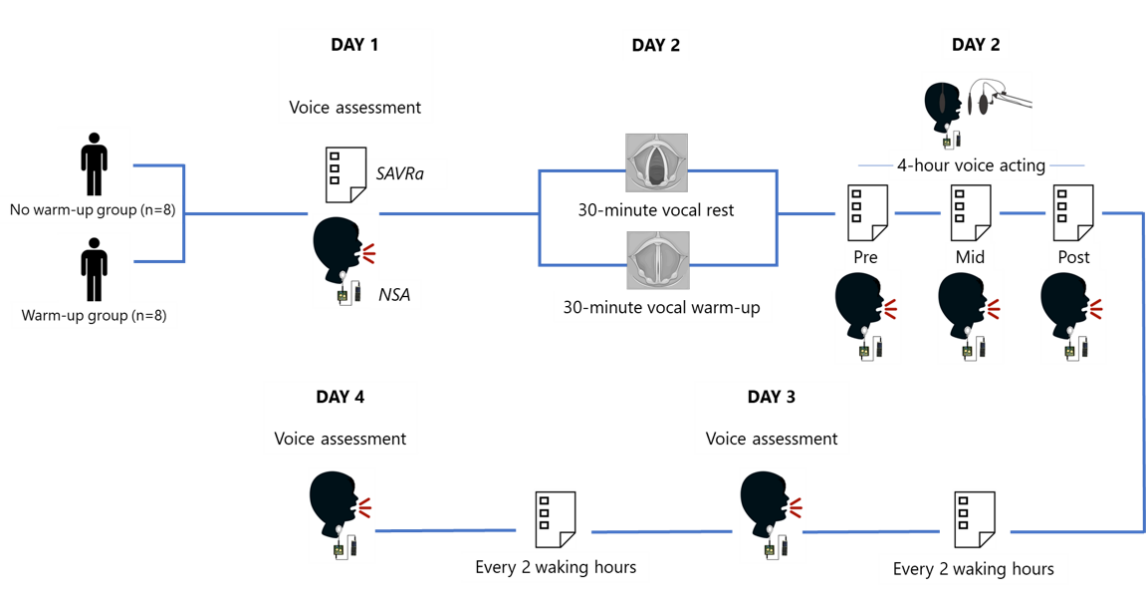
Human Protocol of Voice Assessments and Voice Acting. Voice assessments included Self-Administrated Voice Rating questionnaire (SAVRa) and neck surface accelerometer (NSA)-derived acoustic voice evaluation.

The experimental protocol spanned across 4 consecutive days for various tasks (Figure 2). Voice assessments were conducted on days 1, 3, and 4 at McGill University’s Voice and Upper Airway Research Lab. Voice assessments and a professional voice acting session took place on day 2 at a professional recording studio. Upon arrival to the laboratory or the studio, an NSA was mounted onto a participant’s neck surface around the glottal notch region. Two medical adhesives, (1) a conductive paste on the silicon pad to ensure adherence to the neck skin, and (2) a medical tape, were used to ensure the sensor did not shift during the study.

#### Voice Acting Session

Participants were required to wear an NSA during the whole session. Before the voice acting, the warm-up group participants practiced a 30-minute vocal warm-up routine with a trained speech-language pathologist to ensure the warm-up exercise stayed consistent across participants. The no warm-up group participants were instructed to take vocal rest by refraining from using their voices completely during the 30 minutes preceding the acting session.

After that, all participants proceeded to perform a 4-hour-long voice acting session directed by a professional vocal director. The acting was based on a standardized script from the Assassin’s Creed© video game. Participants were instructed to keep a mouth-to-microphone distance of 50 cm as much as possible without hindering their acting. The air microphone sound was purely used for on-site coaching purpose. Given the confidentiality in video game development, the air microphone data were prohibited for research use.

The acting session consisted of 2 parts: (1) part 1, consisting of low-intensity (eg, casual dialog) voice-over work; and (2) part 2, consisting of medium- (eg, barks, oh-noes) and high-intensity (eg, death cries) voice-over work. The voice director provided feedback to participants on their performance, in an effort to ensure that intensity levels and acting styles were consistent across participants. As a common practice in voice acting, a 15-minute break was provided between parts 1 and 2. Further, participants had access to water and were encouraged to drink throughout sessions. The voice director would reinforce actors to take a drink during sessions when audible “mouth noises” were heard, as the resulting sounds could not be used in the game videos for technical reasons.

#### Voice Assessment Protocol

##### Time Points

The voice assessment protocol included self-perceptual ratings of vocal fatigue and acoustic voice evaluations derived from NSA measurements. The protocol was conducted at 6 study time points: (1) 24 hours before the voice acting session, as a baseline measure; (2) immediately prior to the voice acting session (presession); (3) halfway through the voice acting session (midsession, ie, the 15-minute break between part 1 and part 2 of acting); (4) immediately after the voice acting session (postsession); (5) 24 hours after the voice acting session; and (6) 48 hours after the voice acting session. Participants were also asked to complete the self-perceptual rating questionnaire remotely every 2 waking hours following the voice acting session until the end of the study (Figure 2).

##### Self-Perceptual Ratings of Vocal Fatigue

The Self-Administered Voice Rating (SAVRa) questionnaire was administered to evaluate participants’ perception of vocal fatigue [27]. Three SAVRa ratings were used in this study, namely, *current speaking effort level* (EFFT: 1=no effort, 10=extreme effort to speak), *laryngeal discomfort level* (DISC: 1=no discomfort, 10=extreme discomfort), and *inability to produce soft voice* (IPSV: 1=unproblematic soft voice, 10=extreme problems with producing the soft voice). An electronic version of the SAVRa was created on the SurveyMonkey website [30] for remote data collection.

##### Acoustic Voice Evaluation

To approximate a standard clinical protocol of acoustic voice evaluation, 4 phonation tasks were elicited from participants wearing an NSA (Table 1). A description of the 4 phonation tasks and related acoustic metrics is presented in Table 1.

**Table 1:** Phonation tasks.

Task number	Phonation task	Acoustic metrics
1	1-minute reading of the Rainbow Passage	Cepstral peak prominence Fundamental frequency H1 – H2^a^ Harmonic richness factor Spectral entropy Spectral tilt Surface/skin acceleration level
2	Vowel phonation /a/ for 5 seconds	Cepstral peak prominence Fundamental frequency H1 – H2 Harmonic richness factor Spectral entropy Spectral tilt Surface/skin acceleration level Jitter Shimmer
3	Deep breath and vowel phonation /a/	Maximum phonation time
4	Glide on vowel /a/ from low to high pitch	*f*_0_ minimum *f*_0_ maximum

^a^H1 – H2: difference between the first and second harmonic magnitudes.

Task 1 (Rainbow Passage task) was used to assess acoustic metrics during running speech. Participants were required to read the standard Rainbow Passage for a duration of 1 minute using a pitch, loudness, and pace similar to a natural conversational context. Seven metrics, namely, CPP, *f*_0_, H1 – H2, harmonic richness factor (HRF), spectral entropy (SE), spectral tilt (Tilt), and skin acceleration level (SAL), were extracted during this task.

Task 2 (sustained vowel task) was used to assess acoustic metrics in a more steady-state phonation style. Participants were asked to sustain the vowel sound /a/ for 5 seconds while maintaining a steady pitch and loudness. In addition to the aforesaid metrics, jitter and shimmer were quantified to measure pitch and loudness stability, respectively. Of note, the extraction of jitter and shimmer are only applicable for relatively stable and periodic signals, such as those of sustained vowels herein.

Task 3 (maximum phonation task) was used to measure the maximum time (in seconds) that a person can sustain phonation. Participants were instructed to take a deep breath and produce the vowel /a/ as long as possible, using a comfortable pitch and loudness.

Task 4 (pitch glide task) was used to evaluate an individual’s pitch range. Participants were instructed to start saying /a/ at the lowest pitch possible and slowly glide their voice as high in pitch as possible. Minimum pitch (*f*_0_ minimum) and maximum pitch (*f*_0_ maximum) values were extracted for this task.

### NSA Data Processing

All NSA-related data extraction and calculation were performed using the MATLAB (MathWorks) software. For the computation of acoustic metrics (see Table 2 for detailed algorithms), raw NSA data were first segmented into 45-ms long segments. The voice activity detection method, which was based on short-term energy and zero-crossing rate, was used to remove nonvoiced segments [31]. Only voiced segments were used to extract acoustic metrics and a Hamming window with fast Fourier transform was used to obtain NSA spectra [4]. For CPP, H1 – H2, HRF, Tilt, and SE computation, spectral amplitude normalization was further performed to normalize the amplitudes of all 45-ms spectral segments into the range [0,1]. Furthermore, peak-picking recognition function was applied to identify the harmonics location (ie, H1, H2, H3, ...) for the 4 harmonic-dependent metrics, namely, CPP, H1 – H2, HRF, and Tilt.

**Table 2:** Mathematical formulas and definitions of acoustic metrics.

Acoustic metrics	Mathematic formula	Units	Definition
CPP^a^	Peak_max – (b_0_+b_1_*|q|) where Peak_max is the amplitude in decibels of the highest cepstral peak, b_0_ and b_1_ are the coefficients of the least-square linear regression model of the cepstrum, and q is the quefrency of the highest cepstral peak.	Decibels	The difference in amplitude between the cepstral peak and the corresponding value on the trend line through the overall spectrum, which represents how far the cepstral peak emerges from the cepstrum background.
*f*_0_^b^	1/*T* where T is the period.	Hertz	Frequency of vocal fold vibration that is the lowest of all the frequencies in the voice spectrum and is obtained by the reciprocal of the smallest period.
H1 – H2^c^	20log(A1/A2) where A1 and A2 are the magnitudes of the first and second harmonics in the spectrum, respectively.	Decibels	The log-magnitude difference between the amplitudes of the first and second harmonics in the spectrum.
HRF^d^	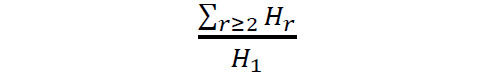 where *H*_*r*_ represents the magnitude of the *r*th harmonic.	Decibels	Ratio of the sum of the amplitudes at the harmonics above the fundamental frequency to the amplitude of the component at the fundamental frequency.
SE^e^	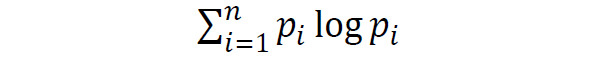 where *p*_*i*_ is the normalized spectral density point (0-3000 Hz).	Relative value	Estimates the uniformity of signal energy distribution in the frequency domain.
Tilt^f^	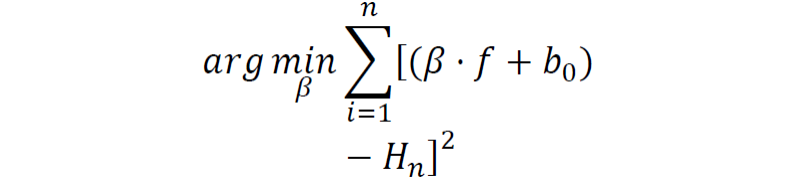 where *H*_*n*_ is the amplitude of spectral harmonics in decibels, *b*_0_ is the least-square linear regression intercept, and *f* is the spectral frequency.	Decibels/Hertz	Tilt of the trend line of the long-term average spectrum, which represents the degree to which intensity drops off as frequency increases.
SAL^g^	20log(max[data_frame]/A_noise) where data_frame is voiced segment and A_noise is the system reference noise level and equal to 0.004 based on the nonvoice waveforms.	Decibels	The calculation is based on the maximum of each voiced segment amplitude for every 45-ms segment window.
Jitter_(relative)_	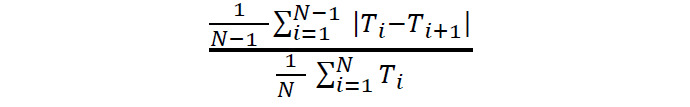 where *T*_*i*_ (i=1, 2, ..., N) is the period of each vocal cycle.	Percent	Average absolute difference between consecutive periods divided by average period, indicating the cycle-to-cycle variation of the fundamental frequency.
Shimmer_(relative)_	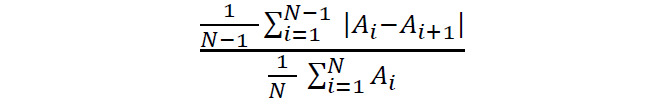 where *A*_*i*_ (i=1, 2, ..., N) is the peak magnitude in each vocal cycle.	Percent	Average absolute difference between the amplitudes of consecutive periods divided by average amplitude, indicating the cycle-to-cycle variation of vocal amplitude.
MPT^h^	T2 – T1 where T2 is the time at which the phonation of a vowel sound finishes and T1 is the time at which the phonation of a vowel sound begins.	Seconds	Measure of a maximally sustained vowel following a maximal inspiration, which provides an indication of the efficiency of the respiratory mechanism.

^a^CPP: cepstral peak prominence.

^b^*f*_0_: fundamental frequency.

^c^H1 – H2: difference between the first and second harmonic magnitudes.

^d^HRF: harmonic richness factor.

^e^SE: spectral entropy.

^f^Tilt: spectral tilt.

^g^SAL: skin acceleration level.

^h^MPT: maximum phonation time.

Conventionally, the computation of these acoustic metrics is based on glottal flow waveforms, which are derived from mouth-radiated acoustic pressure or airflow signals using inverse filtering estimation. However, as the NSA signals are based on skin acceleration, no mouth-radiated pressure components are present for inverse filtering to obtain glottal flow pulses and thus the resulting waveforms. As such, algorithms of H1 – H2, SE, Tilt, and SAL were customized and parameterized in this study [15]. For the calculation of H1 – H2, the first and second harmonics were derived from the NSA spectrum directly. For SE, this metric was computed to quantify the uniformity of signal energy distribution, that is, the degree of chaos, in the frequency domain of the NSA spectrum. From our previously published study [4], the SE was identified as a key acoustic metric in discriminating voice types, in which pressed voice showed higher SE value than those of normal and breathy voice. For the calculation of Tilt, the slope was equal to the amplitude of the spectral harmonics divided by the frequency. In this study, Tilt was computed as a least-square linear regression slope of the long-term average spectrum, which represents the degree to which intensity drops off as frequency increases. The first-order polynomial was used to calculate the slope of the spectral harmonics. For the calculation of SAL, the NSA background noise level was measured as an average value of A_noise, which is equal to 0.004. The SAL was calculated for every 45-ms voiced segments. The SAL was a logarithmic form of the NSA amplitude and showed positive correlation with SPL. Both our own and others work showed that SAL was a good estimate of the SPL outputs in phonation tasks [29,32].

Lastly, for distance dose, the algorithm was based on our previously published work [11]. In brief, equivalent SPL values were first estimated using a logarithmic curve–fitting model on SAL values. The location of each vocal cycle was then identified using the peak-picking recognition function. The equivalent SPL values were used to calculate the oscillating amplitude of vocal folds in each vocal cycle. The oscillating amplitude and the number of vocal cycles were finally used to calculate the total distance that the vocal folds traveled during the recorded time.

### Statistical Analysis

#### Statistical Software

JMP Pro software (version 16.1.0; JMP Statistical Discovery LLC) was used for all statistical analyses. With the high number of contrasts carried out throughout this analysis, a more conservative α value of .01 was used to minimize the chances of a type 1 error.

#### SAVRa Scores

As SAVRa scores were obtained every 2 hours after the acting session, data were reduced by averaging the scores to the corresponding AM or PM of the day. For instance, day 3 scores obtained from 12:00 AM to 11:59 AM were averaged as day 3 AM, whereas those from 12:00 PM to 11:59 PM were averaged as day 3 PM. In addition, individual difference scores were computed for each participant by subtracting mean values at baseline (day 1) from means at each time point and then averaged as described above. Computing and analyzing differences helped to normalize individual variation and allowed for analyses to highlight changes in vocal measurements and fatigue over time. Both means and difference scores were used for statistical analyses.

Mixed-effects ANOVA was performed on each SAVRa score (EFFT, DISC, and IPSV). Either study group (warm-up or no warm-up) or gender group (female or male) was treated as a between-subjects factor in separate mixed-effects ANOVAs. Full-factorial models were not conducted because of the uneven distribution of genders across study groups. *Time* was treated as a within-subjects factor (day 1, day 2 presession, day 2 midsession, day 2 postsession, day 2 PM, day 3 AM, day 3 PM, day 4 AM, day 4 PM). Planned paired contrasts were performed for significant main effects (P<.01), for example, score on each day compared against day 1 (baseline). For analyses involving study group, individual difference scores were used instead of mean values to minimize the effects of the unequal distribution of males and females in each study group.

#### NSA-Derived Distance Dose

Accumulated distance doses for (1) the entire voice acting session (Total Dd), (2) the first part of the session (Dd part 1), and (3) the second part of the session (Dd part 2) were computed for each participant. No data normalization was performed for these data. Mixed-effects ANOVAs were conducted with session dose (Dd part 1 vs Dd part 2) as a within-subjects factor, and study group or gender group as a between-subjects factor. A separate *t* test was conducted for Total Dd.

#### NSA-Derived Acoustic Metrics

For NSA-derived acoustic metrics, mixed-effects ANOVAs were conducted using time as a within-subjects factor (day 1, day 2 presession, day 2 midsession, day 2 postsession, day 3, day 4) and study group or gender group as a between-subjects factor. Planned paired contrasts were performed for significant main effects (P<.01). For analyses involving study group, individual difference scores (magnitude of change compared with baseline) were used instead of mean values.

### Ethical Approval

This human protocol (A04-B21-17A) was approved by the Institutional Review Board at McGill University. The full purpose of the study was not communicated to participants until after completing the study to minimize participant bias on self-perceptual rating measures.

## Results

### Participant Demographics

The breakdown of participant demographics as functions of study group and gender group is shown in Table 3.

**Table 3:** Participant descriptive statistics.

Group			Age (years), mean (SD)		Voice acting experience (years), mean (SD)
**Study group**					
	No warm-up	32 (5.1)		4 (2.9)	
	Warm-up	32 (5.5)		8 (5.7)	
**Gender group**					
	Female	32 (5.5)		7 (5.4)	
	Male	33 (4.7)		4 (3.2)	

### NSA Instrumentation and Analysis Validity

Participants performed their voice acting with NSA wearables for 4 hours in an ecologically valid setting. The wearables stayed in place for all participants regardless of active body movements during the acting session. All planned acoustic metrics were successfully extracted from NSA signals. To further validate the NSA signal processing algorithm, numerical values of our acoustic metrics from the Rainbow Passage task were compared with those extracted from daily conversational speech by other research groups. Our data were found to be within a reasonable numerical range with others, supporting both the ecological and external validity of our instrument and analyses (Table 4).

**Table 4:** Acoustic metrics comparison.^a^

Sources				*f*_0_^b^					CPP^c^			H1 – H2^d^			Tilt^e^			Tilt Abs^f^
				Mode		Mean (SD)		Mean (SD)			Mean (SD)			Mean (SD)			Mean (SD)	
**This study: Rainbow Passage**																		
	**No warm-up group**																	
		T1	104		156.1 (49.0)		20.5 (3.7)			5.4 (17.1)			–0.048 (0.009)			–6.0 (5.3)		
		T2b	108		150.2 (88.0)		26.3 (8.9)			3.8 (17.4)			–0.044 (0.008)			–4.6 (4.0)		
		T3	99		151 (61.4)		29.5 (7.8)			5.2 (18)			–0.041 (0.007)			–6.0 (4.7)		
		T4	81		140.6 (48.8)		26.3 (7.7)			3.8 (16)			–0.045 (0.009)			–4.7 (4.0)		
		T6	85		146.4 (49.3)		21 (3.8)			4.6 (15.6)			–0.049 (0.009)			–4.9 (4.5)		
		T7	82		143 (53.8)		20.7 (3.7)			10.3 (16.2)			–0.049 (0.010)			–5.6 (4.4)		
	**Warm-up group**																	
		T1	176		183.3 (75.8)		21.1 (3.8)			8.2 (23.5)			–0.048 (0.011)			–1.2 (5.0)		
		T2a	173		184.9 (113.3)		23.6 (7.6)			7.4 (21.8)			–0.05 (0.011)			–.07 (5.2)		
		T2b	173		182.5 (76.5)		23.1 (7.6)			8.5 (23.5)			–0.048 (0.011)			–0.8 (4.8)		
		T3	186		182.2 (69.5)		26.7 (8.6)			10.1 (24.8)			–0.045 (0.012)			–1.2 (5.2)		
		T4	138		171.3 (75.0)		24.4 (6.4)			13 (23.3)			–0.045 (0.009)			–2.3 (5.8)		
		T6	151		176.5 (63.9)		21.4 (3.9)			8.7 (23.7)			–0.05 (0.011)			–1.3 (5.2)		
		T7	181		182.4 (70.2)		20.8 (3.9)			4.9 (22)			–0.052 (0.011)			–0.9 (5.7)		
**Van Stan et al [14]: Weeklong summary**																		
	Patients with PVFL^g^			198.1		—^h^ (76.1)		—			—			—			—	
	Matched controls			202.9		— (88.0)		—			—			—			—	
**Mehta et al [10]: Weeklong summary**																		
	Patients with PVH^i^			197.2		— (75.3)		23.2 (4.4)			—			—			–14.4 (2.4)	
	PVH controls			201.4		— (89.6)		22.9 (4.5)			—			—			–14.1 (2.4)	
	Patients with NPVH^j^			193.8		— (73.5)		21.4 (4.2)			—			—			–13.6 (2.5)	
	NPVH controls			192.9		— (70.1)		22.8 (4.4)			—			—			–14.1 (2.4)	
**Van Stan et al [18]: Weeklong summary**																		
	Patients with PVH			196.1		— (73.5)		23.1 (4.4)			4.4 (6.1)			—			—	
	Matched controls			199.4		— (86.7)		22.7 (4.4)			5.1 (7.0)			—			—	
**Toles et al [33]: Weeklong summary**																		
	Combined phonation (healthy)			205.7		— (91.6)		22.7 (4.5)			5.5 (7.2)			—			—	
	Singing (healthy)			325.4		— (94.6)		21.5 (4)			9.7 (7.3)			—			—	
	Speech (healthy)			203.5		— (62.4)		23.1 (4.5)			4.2 (6.6)			—			—	
**Van Stan et al [34]: Weeklong summary**																		
	Patients with NPVH			202.4		— (68.1)		20.6 (3.9)			2.6 (6.7)			—			—	
	Matched controls			182.8		— (68.6)		22.1 (4.3)			2.5 (6.5)			—			—	

^a^Mode and mean (SD) data for the acoustic metrics *f*_0_, CPP, H1 – H2, Tilt, and Tilt Abs are presented for our Rainbow Passage task as well as for conversational speech from related research studies.

^b^*f*_0_: fundamental frequency.

^c^CPP: cepstral peak prominence.

^d^H1 – H2: difference between the first and second harmonic magnitudes.

^e^Tilt: spectral tilt.

^f^Tilt Abs: tilt absolute.

^g^PVFL: phonotraumatic vocal fold lesions.

^h^—: data not available.

^i^PVH: phonotraumatic vocal hyperfunction.

^j^NPVH: nonphonotraumatic vocal hyperfunction.

### SAVRa

**Figure 3:**
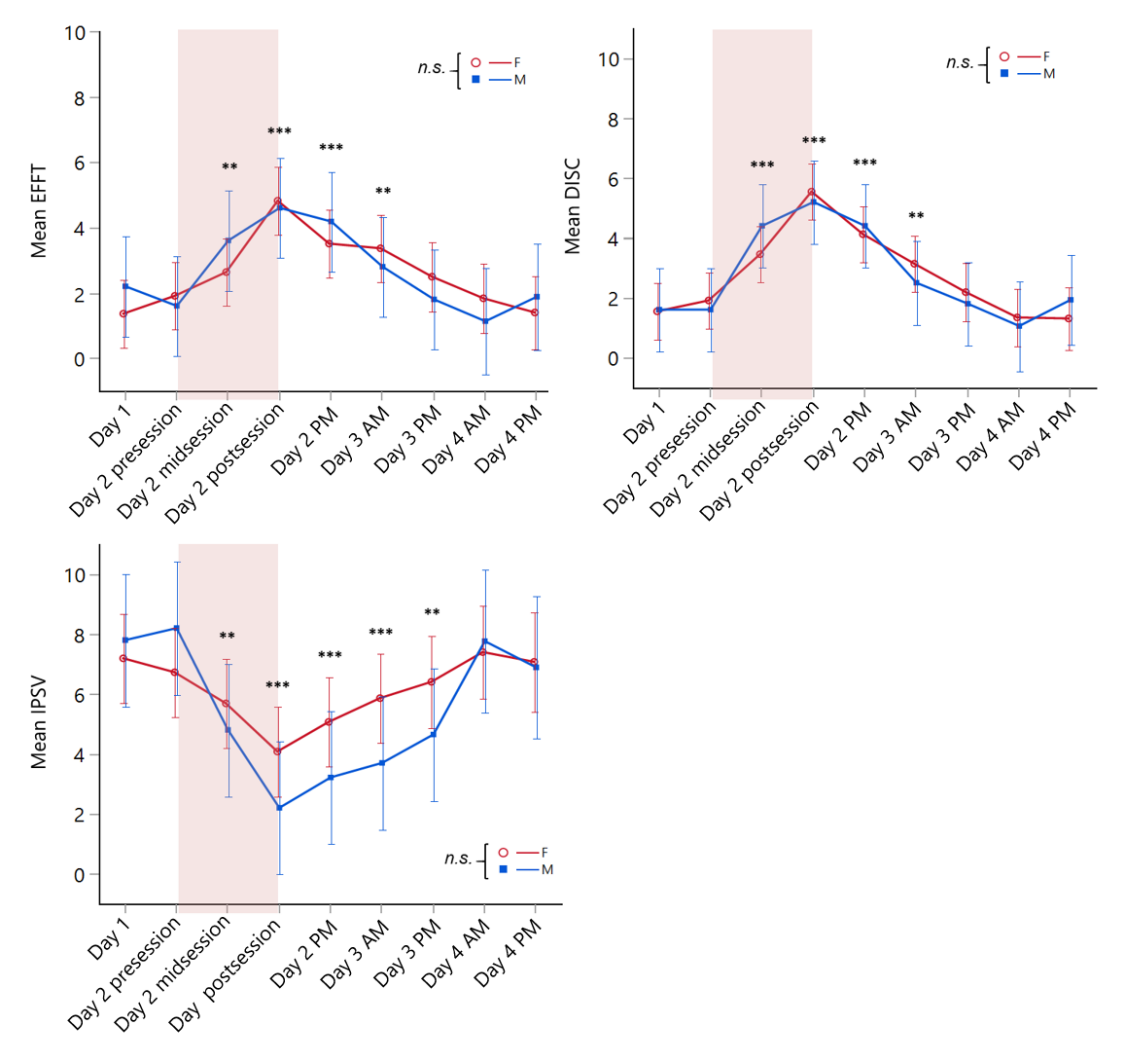
Means and standard errors (error bars) of Self-Administrated Voice Rating questionnaire (SAVRa) as functions of Time and Gender Group. The voice acting session is highlighted in the pink region. Asterisks denote statistically significant differences between a specific time point and Day 1 (**P≤.01, *** P≤.001). DISC: laryngeal discomfort level; EFFT: current speaking effort level; IPSV: inability to produce soft voice; n.s.=no significant differences.

No significant effects of study group or gender group were observed for SAVRa measures, but a main effect of time was found on all 3 SAVRa scores (all P<.001; Figure 3; see Multimedia Appendices 1 and 2 for detailed test statistics). Post hoc tests showed that EFFT and DISC scores were all significantly higher than baseline from day 2 midsession to day 3 AM (all P<.01). IPSV scores were significantly lower than baseline starting from day 2 midsession to day 3 PM (all P<.01). These results suggest that professional voice acting could induce self-perceived vocal fatigue as early as 2 hours after the start of acting, with potential recovery occurring 24-48 hours after the completion of acting session.

### NSA-Derived Distance Dose

**Figure 4:**
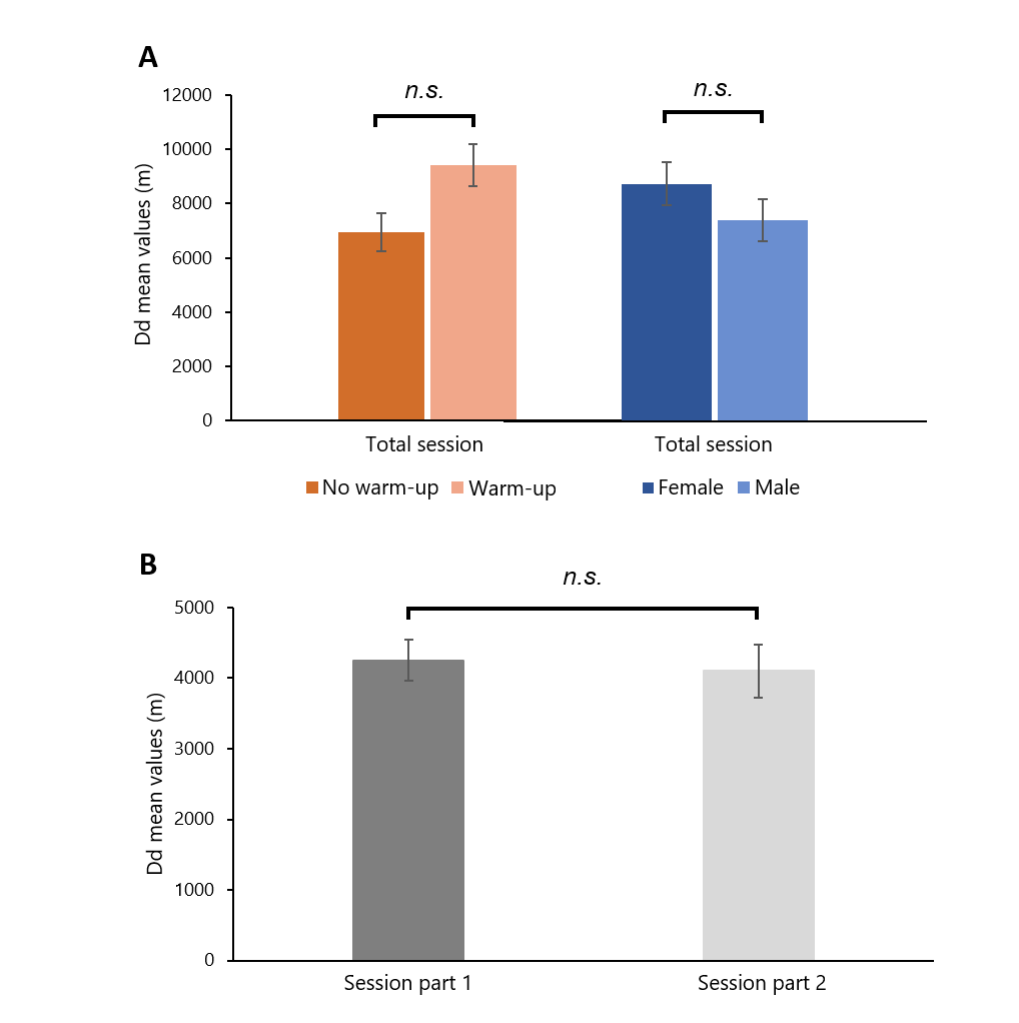
Means and standard errors (error bars) of accumulated distance dose (Dd) as functions of Study Group and Gender Group. (A) Total 4-hour sessions. (B) First and second parts of session. n.s.=no significant differences, ie, *P*>.01.

No significant main effects of study group, gender group, or session dose were found on independent tests of distance dose. The averaged Total Dd was approximately 8354.35 m (SD 2301.84 m) for a 4-hour voice acting across participants. The averaged Dd was approximately 4250.24 m (SD 1408.31 m) for part 1 and 4104.11 m (SD 1086.22 m) for part 2 of the acting session (Figure 4; see Multimedia Appendix 3 for detailed test statistics).

### NSA-Derived Acoustic Metrics

#### Overview of Tasks

Across all phonation tasks, no study group effects (ie, main effect of study group or interaction of study group and time) were noted for acoustic metrics. By contrast, main and interaction effects of gender group and time were found in certain acoustic metrics depending on the phonation task.

#### Rainbow Passage Task

**Figure 5:**
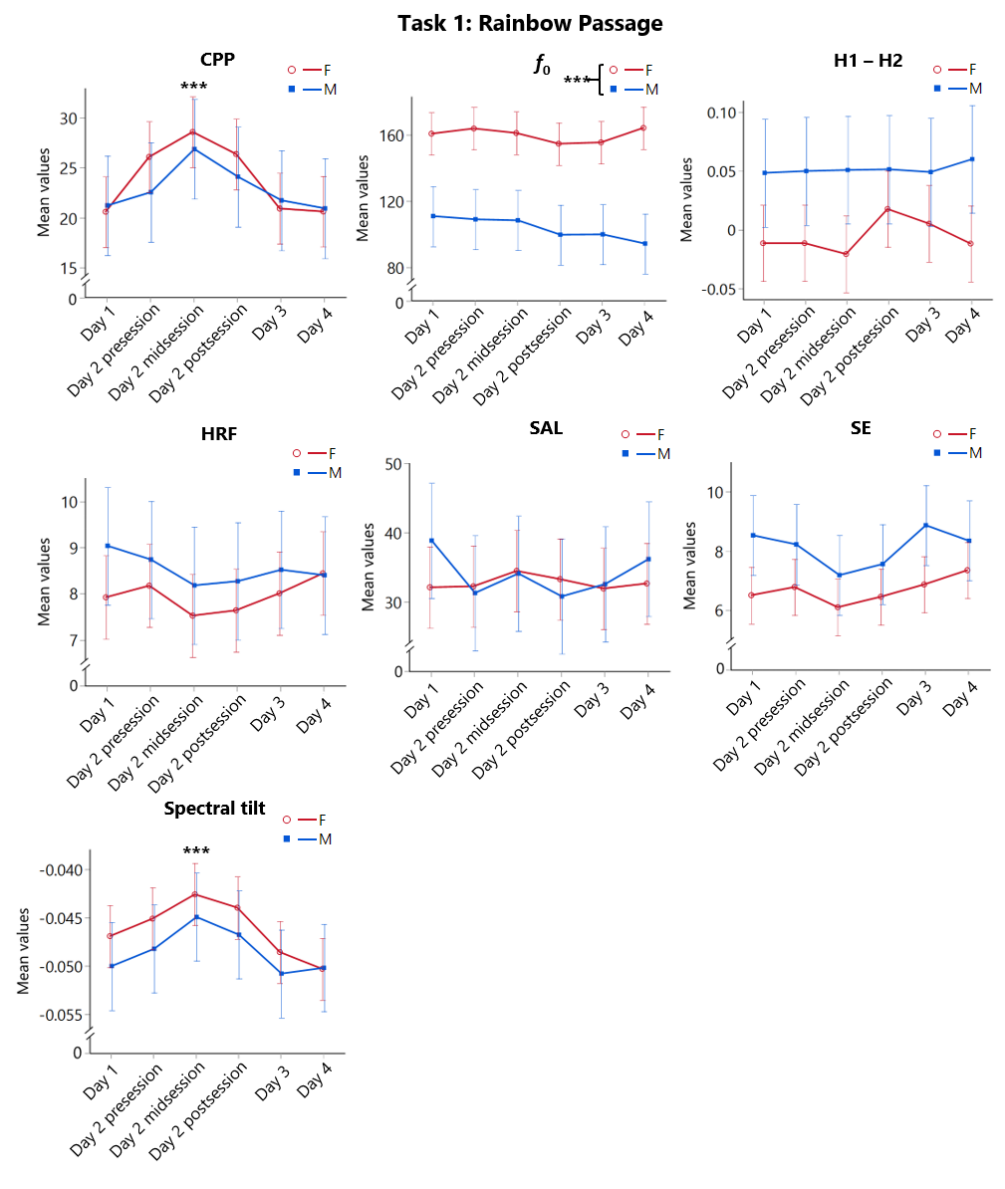
Means and standard errors (error bars) of neck surface accelerometer-derived acoustic metrics in the Rainbow Passage Task as functions of Time and Gender Group. Asterisks denote statistically significant differences (1) between the female (F) and the male (M) participant groups, as well as, (2) between a specific time point and Day 1 (*** P≤.001). CPP: cepstral peak prominence; *f*_0_: fundamental frequencyo; H1 – H2: difference between the first and second harmonic magnitudes; HRF: harmonic richness factor; SAL: skin acceleration level; SE: spectral entropy.

There was a main effect of time for CPP and Tilt (both P<.001) measures, but no significant gender group or interaction effects (Figure 5; see Multimedia Appendices 4 and 5 for detailed test statistics). Both measures followed a similar trajectory as those observed in SAVRa, with values increasing from day 1 to day 2 midsession and then decreasing thereafter. Post hoc tests showed that values at day 2 midsession were significantly greater than baseline values (day 1) for both measures (CPP: P<.001; Tilt: P=.001). Testing also yielded a significant main effect of gender group for *f*_0_ (P<.001), with females demonstrating higher *f*_0_ values throughout. The gender difference on *f*_0_ was expected because females generally have higher conversational pitches than males in vocally healthy populations. No significant gender group, time, or interaction effects were found in other acoustic metrics (see Multimedia Appendix 4 for detailed test statistics).

#### Sustained Vowel Task

**Figure 6:**
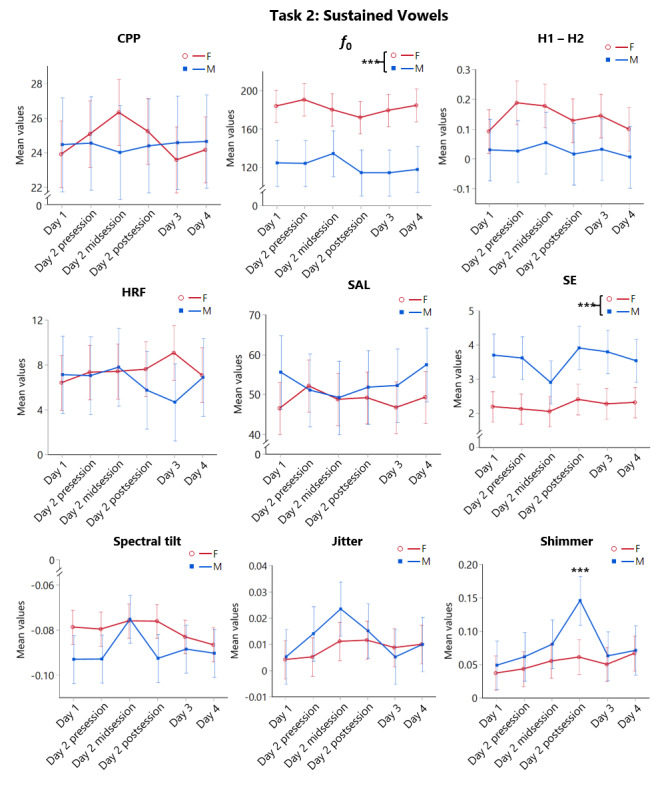
Means and standard errors (error bars) of neck surface accelerometer-derived acoustic metrics in the Sustained Vowel Task as functions of Time and Gender Group. Asterisks denote statistically significant differences (1) between the female (F) and the male (M) participant groups, as well as, (2) between a specific time point and Day 1 (*** P≤.001). CPP: cepstral peak prominence; *f*_0_: fundamental frequencyo; H1 – H2: difference between the first and second harmonic magnitudes; HRF: harmonic richness factor; SAL: skin acceleration level; SE: spectral entropy.

There was a significant main effect of time for shimmer (P<.01), with values peaking at day 2 postsession and then dropping off afterward (Figure 6; see Multimedia Appendices 6 and 7 for detailed test statistics). Post hoc tests indicated that shimmer values at day 2 postsession were significantly higher than baseline values (P=.001). This appeared to be driven by the male group, whose overall values at day 2 postsession were higher than the female group; however, effects of gender group did not reach significance (see Multimedia Appendix 6 for detailed test statistics). The main effects of gender group were also noted for SE and *f*_0_ (both P<.001). For SE, males had higher values throughout, while females showed higher values throughout for *f*_0_. No significant gender group, time, or interaction effects were found in other acoustic metrics (see Multimedia Appendix 6 for detailed test statistics).

#### Maximum Phonation Time and Pitch Glide Tasks

No effects of gender group, time, or their interaction were noted for maximum phonation time (MPT), *f*_0_ minimum, and *f*_0_ maximum (Table 5).

**Table 5:** Group-based means (SD) for the maximum phonation time and pitch glide tasks.^a^

Acoustic metrics and gender groups			Experimental time points, mean (SD)													ANOVA				
			Day 1		Day 2 presession		Day 2 midsession		Day 2 postsession		Day 3		Day 4		Time			Gender		Time × gender
**MPT^b^**															*F*_5,65_=1.38; P=.25			*F*_1,13_=2.37; P=.24		*F*_5,65_=0.81; P=.54
	Female	25.29 (7.53)		22.60 (6.42)		25.68 (6.84)		24.12 (7.38)		22.22 (6.86)		24.90 (7.90)								
	Male	30.08 (8.51)		26.74 (11.35)		30.41 (15.36)		30.56 (10.65)		30.87 (12.11)		28.59 (10.50)								
***f*_0_ min^c^**															*F*_5,65_=3.02; P=.02			*F*_1,13_=3.65; P=.08		*F*_5,65_=3.24; P=.011
	Female	13.98 (4.88)		12.85 (0.41)		12.83 (0.62)		12.87 (0.42)		15.03 (7.62)		12.63 (0.61)								
	Male	12.77 (0.68)		16.80 (8.80)		14.06 (3.57)		13.08 (0.56)		18.81 (8.43)		25.18 (16.20)								
***f*_0_ max^d^**															*F*_5,65_=0.84; P=.53			*F*_1,13_=2.37; P=.15		*F*_5,65_=1.57; P=.18
	Female	929.14 (335.34)		870.47 (269.30)		911.29 (211.76)		934.05 (301.39)		842.46 (242.24)		864.20 (231.92)								
	Male	694.61 (259.44)		689.70 (281.84)		661.19 (188.34)		626.75 (191.97)		823.09 (614.29)		574.22 (249.39)								

^a^There are no statistically significant effects (P<.01).

^b^MPT: maximum phonation time.

^c^*f*_0_ min: *f*_0_ minimum.

^d^*f*_0_ max: *f*_0_ maximum.

## Discussion

### Principal Findings and Comparison With Prior Work

Accumulated distance doses are used to estimate a person’s voice use [24,25]. Individuals with healthy voices were reported to have accumulated distance doses of around 18,000 m/week and 228 m/hour [10]. For individuals with disordered voices, the numbers were found to be notably higher with around 27,000 m/week and 345 m/hour [10,14]. In this study, for a total of 4-hour typical voice acting, accumulated distance doses were 8354.35 m on average, with approximately 2089 m/hour. Compared with the literature data, voice actors who engaged in 4 hours of voice acting in this study accumulated almost 46% of a typical person’s weekly voice use (8354/18,000, 46.41%). In real-world situations, professional voice actors are often booked with more than 1 acting session in a week, suggesting an exceptionally high vocal demand at the acting workplace. A recent study further investigated the vocal doses from singers with vocal injury in their regular weeks [33]. Results showed that most distance doses in these singers were associated with speaking voice (about 268 m/hour) rather than singing voice (about 103 m/hour) in their weekly summaries (about 370 m/hour). Taken together, these results suggest the need for continuous voice monitoring in voice actors and other occupational voice users, not only in the workplace but also in daily life, to support further self-awareness and management of safe voice use.

Based on the SAVRa data, participants started to perceive significant increases in vocal effort and discomfort after the first part of the acting. The scores increased during acting, reached their peak right after acting, and gradually returned to baseline within 48 hours after acting. This arc-shaped trajectory replicated the same SAVRa variations obtained from our previous vocal loading study, in which participants were required to reach a distance dose of 500 m in each of the 6 consecutive voice sessions [11]. Among all NSA-derived metrics across phonation tasks, only *CPP* and *Tilt* from the Rainbow Passage most closely mirrored the temporal trends of the SAVRa with significant changes over time in both genders. These results are encouraging as *CPP* is already regarded as a robust measure of vocal fatigue and voice deviation with air acoustic microphone signals [35,36]. Even though our NSAs have more restricted bandwidth (around 3 kHz), the clinical robustness of CPP seemed to be preserved. For *Tilt,* a decreased slope of Tilt is suggested to correlate with perceived creaky voice, whereas an increased slope can be associated with breathy voice [37,38]. Our results showed that the *Tilt* measure increased with the time of voice acting. Individuals may tend to deviate from their modal voice type to a breathier phonation with the vocal fatigue ensued from acting. Overall, results from our current and previous study [11] agreed that, among all NSA acoustic metrics, *CPP* and *Tilt* were most robust to reflect an individual’s voice variations and vocal fatigue.

In sum, both female and male actors showed comparable accumulated distance doses from voice acting, suggesting a gender-specific vocal safety limit may not be necessary. Similar to the observation from air microphone signals, NSA-derived acoustic metrics performed differently between sustained vowels and running speech, whereby the latter is more ecologically valid [19]. In particular, NSA-derived *CPP* and *Tilt* from running speech were equally robust for the detection of voice variations in both genders. These 2 NSA metrics can thus be used as universal surrogates of vocal health biomarkers. One key application of this NSA wearable is to prompt occupational voice users when their vocal safety limits are reached for duly protection. However, continuous, real-time monitoring of an individual’s body sound signals requires substantial computing power. Algorithms can thus be focused on processing selected metrics that are the most clinically relevant, such as accumulated distance dose, *CPP*, and *Tilt*. Machine learning techniques can be further applied to learn the time history of an individual’s voice features, capture their detrimental variations, and predict risk levels of vocal injury. This functionality will enable continuous self-awareness of vocal behavior and protection of vocal safety in occupational voice users.

### Limitations and Future Directions

The NSA system used in this study was a wired version, which poses challenges for users to wear it for long periods. The data transfer was also through a physical recorder and then to a personal computer. No user-device interaction such as biofeedback of voice use was built into the current NSA system. To address these issues critical to mHealth, a wireless version of NSA wearable is now under development in our group. The NSA data will be transmitted through Bluetooth low-energy technology to a smartphone device. An in-house mobile app is also in development with features of NSA data visualization and vocal health feedback. We have already developed machine learning algorithms that are lean and efficient enough to classify upper airway symptoms such as cough and throat clearing on the NSA board [5]. The aforesaid system upgrades will broaden the NSA functionality to be more interactive and suitable for all-day monitoring.

### Conclusions

Laboratory NSA wearable devices were deployed to a group of professional voice actors who underwent a 4-hour voice acting session. The devices were able to tolerate the strenuous body movements and ensued body movement noise from voice acting. Vocal dose measures and a regular check of clinical evaluation metrics (SAVRa and NSA-derived acoustic metrics) were included in this investigation to validate the instrumentation of the device, the NSA-derived acoustic metrics, and NSA’s algorithm for voice monitoring. Future field tests are warranted to evaluate aforesaid new instrument and algorithm functions in predicting voice and airway health for occupational voice users and those with chronic airway diseases.

## Appendix

Multimedia Appendix 1 Group-based means for SAVRa scores. Means (standard deviation) for each SAVRa item are presented for females and males across time points. F-values, degrees of freedom, and *P* values and from ANOVA testing are also reported for each factor (Time, Gender) and their interaction (Time x Gender). Statistically significant effects (*P*<.01) are indicated in bold.

Multimedia Appendix 2 Post hoc testing results for SAVRa scores. t Ratios, *P* values and Cohen’s d effect sizes are presented for post hoc analyses of significant main effects of Time. For each SAVRa item, planned paired contrasts comparing scores at each time point against Day 1 (baseline) were conducted. Statistically significant effects (*P*<.01) are indicated in bold.

Multimedia Appendix 3 Group-based means for Distance Dose measures. Means (standard deviation) for each distance dose measure are presented by Study Group (No Warm-Up and Warm-Up) and Gender Group (Females and Males). F-values, degrees of freedom, and *P* values from ANOVA testing are reported for each factor (Session Dose, Study Group, Gender) and their interaction (Time x Study Group, Time x Gender). t-values, degrees of freedom, and *P* values from t-testing are also reported. There are no statistically significant effects (*P*<.01).

Multimedia Appendix 4 Group-based means for the Rainbow Passage task. Means (standard deviation) for each voice metric are presented for females and males across time points. F-values, degrees of freedom, and *P* values from ANOVA testing are also reported for each factor (Time, Gender) and their interaction (Time x Gender). Statistically significant effects (*P*<.01) are indicated in bold.

Multimedia Appendix 5 Post hoc testing results for the Rainbow Passage task. t Ratios, *P* values and Cohen’s d effect sizes are presented for post hoc analyses of acoustic measures showing significant main effects of Time (CPP and Tilt) and Gender (*f*_0_). For main effects of Time, planned paired contrasts comparing scores at each time point against Day 1 (baseline) were conducted. For the main effect of Gender, Female values were compared against Male values. Statistically significant effects (*P*<.01) are indicated in bold.

Multimedia Appendix 6 Group-based means for the Sustained Vowel task. Means (standard deviation) for each voice metric are presented for females and males across time points. F-values, degrees of freedom, and *P* values from ANOVA testing are also reported for each factor (Time, Gender) and their interaction (Time x Gender). Statistically significant effects (*P*<.01) are indicated in bold.

Multimedia Appendix 7 Post hoc testing results for the Sustained Vowel task. t Ratios, *P* values and Cohen’s d effect sizes are presented for post hoc analyses of acoustic measures showing significant main effects of Time (Shimmer) and Gender (*f*_0_ and SE). For the main effect of Time, planned paired contrasts comparing scores at each time point against Day 1 (baseline) were conducted. For main effects of Gender, Female values were compared against Male values. Statistically significant effects (*P*<.01) are indicated in bold.

## Abbreviations

ACTRA: Alliance of Canadian Cinema, Television and Radio Artists
CPP: cepstral peak prominence
CRBLM: Centre for Research on Brain, Language and Music Research
Dd: distance dose
DISC: laryngeal discomfort level
EFFT: current speaking effort level
*f*_0_: fundamental frequency
H1 – H2: difference between the first and second harmonic magnitudes
HRF: harmonic richness factor
IPSV: inability to produce soft voice
mHealth: mobile health
MPT: maximum phonation time
NSA: neck surface accelerometer
SAL: skin acceleration level
SAVRa: Self-Administered Voice Rating questionnaire
SE: spectral entropy
SPL: sound pressure level
Tilt: spectral tilt
