# Supplementary material for: Wearable Neck Surface Accelerometers for Occupational Vocal Health Monitoring: Instrument and Analysis Validation Study
Source: JMIR Form Res. 2022 Aug 5;6(8):e39789. doi: 10.2196/39789 (PMC9391979; doi:10.2196/39789)
Supplement: Multimedia Appendix 1 [file formative_v6i8e39789_app1.docx]

| Item | Gender Group | Experimental Time Points – M (SD) | | | | | | | | | ANOVA | | |
| --- | --- | --- | --- | --- | --- | --- | --- | --- | --- | --- | --- | --- | --- |
|  |  | Day 1 | Day 2 pre-session | Day 2 mid-session | Day 2 post-session | Day 2 p.m. | Day 3 a.m. | Day 3 p.m. | Day 4 a.m. | Day 4 p.m. | Time | Gender | Time x Gender |
| EFFT^a^ | Female | 1.36 (0.67) | 1.91 (1.30) | 2.64 (1.03) | 4.82 (2.68) | 3.51 (2.01) | 3.36 (1.89) | 2.66 (1.53) | 2.00 (1.13) | 1.46 (0.61) | *F_(8,105)_*=11.67  ***P*<.001** | *F_(1,13.83)_*=.006  *P*=.94 | *F_(8,105)_*=1.07  *P*=.39 |
|  | Male | 2.20 (1.30) | 1.60 (0.89) | 3.60 (2.79) | 4.60 (2.79) | 4.18 (2.41) | 2.80 (1.48) | 1.80 (0.77) | 1.50 (0.58) | 2.25 (1.89) |  |  |  |
| DISC^b^ | Female | 1.55 (0.82) | 1.91 (1.22) | 3.45 (1.57) | 5.55 (2.42) | 4.12 (2.04) | 3.14 (1.47) | 2.36 (1.38) | 1.52 (0.82) | 1.44 (0.61) | *F_(8,104.7)_*=18.91  ***P*<.001** | *F_(1,13.46)_*=.000  *P*=.99 | *F_(8,104.7)_*=.61  *P=*.77 |
|  | Male | 1.60 (0.89) | 1.60 (0.89) | 4.40 (2.07) | 5.20 (2.39) | 4.40 (2.22) | 2.50 (1.12) | 1.80 (0.77) | 1.38 (0.48) | 2.25 (1.89) |  |  |  |
| IPSV^c^ | Female | 7.19 (3.00) | 6.73 (2.72) | 5.69 (3.14) | 4.08 (2.82) | 5.07 (2.62) | 5.86 (2.63) | 6.09 (1.82) | 7.08 (2.15) | 6.81 (2.14) | *F_(8,104.8)_*=9.98  ***P* <.001** | *F_(1,13.56)_*=.56  *P*=.47 | *F_(8,104.8)_*=1.65  *P*=.12 |
|  | Male | 7.80 (2.28) | 8.20 (2.05) | 4.80 (3.11) | 2.20 (1.79) | 3.22 (0.82) | 3.70 (1.57) | 4.65 (1.12) | 7.63 (0.48) | 6.75 (1.89) |  |  |  |

**Table S1.** **Group-based means for SAVRa scores.** Means (standard deviation) for each SAVRa item are presented for females and males across time points. F-values, degrees of freedom, and P-values and from ANOVA testing are also reported for each factor (Time, Gender) and their interaction (Time x Gender). Statistically significant effects (p < .01) are indicated in **bold.**

^a^EFFT=Current speaking effort level; ^b^DISC=Laryngeal discomfort; ^c^IPSV=Inability to produce soft voice
