# Supplementary material for: Wearable Neck Surface Accelerometers for Occupational Vocal Health Monitoring: Instrument and Analysis Validation Study
Source: JMIR Form Res. 2022 Aug 5;6(8):e39789. doi: 10.2196/39789 (PMC9391979; doi:10.2196/39789)
Supplement: Multimedia Appendix 2 [file formative_v6i8e39789_app2.docx]

| Time Points | EFFT^a^ | | | DISC^b^ | | | IPSV^c^ | | |
| --- | --- | --- | --- | --- | --- | --- | --- | --- | --- |
|  | *t* Ratio | Prob>\|*t*\| | Cohen's *d* | *t* Ratio | Prob>\|*t*\| | Cohen's *d* | *t* Ratio | Prob>\|*t*\| | Cohen's *d* |
| Day 1 x Day 2 pre-session | 0.06 | .95 | 0.18 | -0.39 | .70 | 0.26 | 0.05 | .96 | 0.07 |
| Day 1 x Day 2 mid-session | -2.93 | **<.001** | 0.94 | -5.07 | **<.001** | 1.62 | 3.15 | **<.001** | 0.68 |
| Day 1 x Day 2 post-session | -6.42 | **<.001** | 1.58 | -8.18 | **<.001** | 2.21 | 6.09 | **<.001** | 1.45 |
| Day 1 x Day 2 p.m. | -4.53 | **<.001** | 1.29 | -5.79 | **<.001** | 1.71 | 4.69 | **<.001** | 1.13 |
| Day 1 x Day 3 a.m. | -2.85 | **.01** | 1.11 | -2.68 | **0.01** | 1.22 | 3.79 | **<.001** | 0.83 |
| Day 1 x Day 3 p.m. | -0.80 | .43 | 0.64 | -0.90 | .37 | 0.59 | 2.73 | **.01** | 0.77 |
| Day 1 x Day 4 a.m. | 0.62 | .54 | 0.24 | 0.76 | .45 | 0.11 | -0.11 | .91 | 0.06 |
| Day 1 x Day 4 p.m. | 0.29 | .78 | 0.09 | -0.09 | .93 | 0.14 | 0.67 | .51 | 0.25 |

**Table S2. Post hoc testing results for SAVRa scores.** t Ratios, P values and Cohen’s d effect sizes are presented for post hoc analyses of significant main effects of Time. For each SAVRa item, planned paired contrasts comparing scores at each time point against Day 1 (baseline) were conducted. Statistically significant effects (P<.01) are indicated in **bold.**

^a^EFFT=Current speaking effort level; ^b^DISC=Laryngeal discomfort; ^c^IPSV=Inability to produce soft voice
