# Supplementary material for: Wearable Neck Surface Accelerometers for Occupational Vocal Health Monitoring: Instrument and Analysis Validation Study
Source: JMIR Form Res. 2022 Aug 5;6(8):e39789. doi: 10.2196/39789 (PMC9391979; doi:10.2196/39789)
Supplement: Multimedia Appendix 3 [file formative_v6i8e39789_app3.docx]

| Study Group | Distance Dose Measures - M (SD) | | ANOVA | | |
| --- | --- | --- | --- | --- | --- |
|  | Dd Part 1 | Dd Part 2 | Session Dose | Study Group | Session Dose x Study Group |
| No Warm-Up | 3503.20  (917.68) | 3448.53  (980.45) | *F_(1,12)_*=.23  *P*=.64 | *F_(1,12)_*=5.14  *P*=.04 | *F_(1,12)_*=.08  *P*=.78 |
| Warm-Up | 4810.53  (1498.24) | 4595.79  (927.30) |  |  |  |
|  | | | | | |
| Gender Group | Dd Part 1 | Dd Part 2 | Session Dose | Gender | Session Dose x Gender |
| Female | 4469.74  (1587.05) | 4266.42  (1079.87) | *F_(1,12)_*=.11  *P*=.75 | *F_(1,12)_*=.96  *P*=.35 | *F_(1,12)_*=.10  *P*=.75 |
| Male | 3701.51  (690.09) | 3698.34  (1143.27) |  |  |  |
|  | | | | | |
| Study Group | Total Dd – M (SD) | | T-Test (Two-Tailed) | | |
| No Warm-Up | 6951.73  (1732.36) | | *t_12_* =2.27  *P*=.04 | | |
| Warm-Up | 9406.32  (2178.38) | |  |  |  |
|  | | | | | |
| Gender Group | Total Dd – M (SD) | | T-Test (Two-Tailed) | | |
| Female | 8736.15  (2504.90) | | *t_8_* =1.25  *P*=0.25 | | |
| Male | 7399.85  (1560.66) | |  |  |  |

**Table S3. Group-based means for Distance Dose measures.** Means (standard deviation) for each distance dose measure are presented by Study Group (No Warm-Up and Warm-Up) and Gender Group (Females and Males). F-values, degrees of freedom, and P-values from ANOVA testing are reported for each factor (Session Dose, Study Group, Gender) and their interaction (Time x Study Group, Time x Gender). t-values, degrees of freedom, and P-values from t-testing are also reported. There are no statistically significant effects (P<.01).
