# Supplementary material for: Wearable Neck Surface Accelerometers for Occupational Vocal Health Monitoring: Instrument and Analysis Validation Study
Source: JMIR Form Res. 2022 Aug 5;6(8):e39789. doi: 10.2196/39789 (PMC9391979; doi:10.2196/39789)
Supplement: Multimedia Appendix 4 [file formative_v6i8e39789_app4.docx]

**Table S4. Group-based means for the Rainbow Passage task.** Means (standard deviation) for each voice metric are presented for females and males across time points. F-values, degrees of freedom, and P-values from ANOVA testing are also reported for each factor (Time, Gender) and their interaction (Time x Gender). Statistically significant effects (P<.01) are indicated in **bold.**

| Feature | Gender Group | Experimental Time Points – M (SD) | | | | | | ANOVA | | |
| --- | --- | --- | --- | --- | --- | --- | --- | --- | --- | --- |
|  |  | Day 1 | Day 2 pre-session | Day 2 mid-session | Day 2 post-session | Day 3 | Day 4 | Time | Gender | Time x Gender |
| CPP | Female | 20.58 (1.11) | 26.09 (9.55) | 28.60 (8.40) | 26.38 (6.01) | 20.91 (1.98) | 20.61 (0.99) | *F_(5,65)_*=4.78  ***P*<.001** | *F_(1,13)_*=.25  *P*=.63 | *F_(5,65)_*=.48  *P*=.79 |
|  | Male | 21.21 (0.57) | 22.55 (5.09) | 26.88 (8.17) | 24.10 (7.47) | 21.72 (1.20) | 20.92 (1.49) |  |  |  |
| F0 | Female | 160.68 (18.25) | 163.82 (20.88) | 161.09 (21.41) | 154.64 (22.10) | 155.42 (20.04) | 164.19 (18.41) | *F_(5,65)_*=3.15  *P*=.013 | *F_(1,13)_*=33.96  ***P*<.001** | *F_(5,65)_*=2.12  *P*=.08 |
|  | Male | 110.87 (14.37) | 108.96 (17.36) | 108.35 (17.41) | 99.65 (11.64) | 99.86 (22.46) | 94.28 (18.52) |  |  |  |
| H1-H2 | Female | -0.011 (0.055) | -0.011 (0.050) | -0.021 (0.054) | 0.018 (0.061) | 0.005 (0.055) | -0.012 (0.058) | *F_(5,65)_*=.62  *P*=.69 | *F_(1,13)_*=6.66  *P*=.02 | *F_(5,65)_*=.73  *P*=.61 |
|  | Male | 0.049 (0.031) | 0.050 (0.040) | 0.051 (0.040) | 0.052 (0.040) | 0.049 (0.022) | 0.060 (0.028) |  |  |  |
| HRF | Female | 7.92 (1.50) | 8.17 (1.72) | 7.52 (1.51) | 7.64 (1.74) | 8.01 (1.53) | 8.44 (1.30) | *F_(5,65)_*=.84  *P*=.53 | *F_(1,13)_*=1.08  *P*=.32 | *F_(5,65)_*=.39  *P*=.85 |
|  | Male | 9.04 (0.57) | 8.74 (1.20) | 8.18 (1.16) | 8.27 (1.31) | 8.52 (0.51) | 8.40 (1.01) |  |  |  |
| SE | Female | 6.51 (1.55) | 6.79 (1.35) | 6.11 (1.53) | 6.46 (1.48) | 6.88 (1.70) | 7.36 (1.16) | *F_(5,65)_*=2.05  *P*=.08 | *F_(1,13)_*=6.95  *P*=.02 | *F_(5,65)_*=.48  *P*=.79 |
|  | Male | 8.54 (1.37) | 8.23 (2.32) | 7.19 (1.69) | 7.57 (1.73) | 8.88 (0.62) | 8.35 (1.21) |  |  |  |
| Tilt | Female | -0.047 (0.004) | -0.045 (0.007) | -0.043 (0.006) | -0.044 (0.004) | -0.0486 (0.0049) | -0.050 (0.004) | *F_(5,65)_*=6.67  ***P*<.001** | *F_(1,13)_=*1.17  *P*=.30 | *F_(5,65)_*=.38  *P*=.86 |
|  | Male | -0.050 (0.003) | -0.048 (0.005) | -0.045 (0.007) | -0.047 (0.005) | -0.051 (0.003) | -0.050 (0.004) |  |  |  |
| SAL | Female | 32.13 (8.22) | 32.27 (9.94) | 34.50 (9.82) | 33.31 (8.48) | 31.96 (7.64) | 32.68 (9.03) | *F_(5,65)_=*.80  *P*=.55 | *F_(1,13)_*=.09  *P*=.76 | *F_(5,65)_*=.95  *P*=.45 |
|  | Male | 38.88 (8.80) | 31.31 (10.37) | 34.12 (5.36) | 30.82 (10.42) | 32.60 (12.49) | 36.20 (8.45) |  |  |  |
