# Supplementary material for: Wearable Neck Surface Accelerometers for Occupational Vocal Health Monitoring: Instrument and Analysis Validation Study
Source: JMIR Form Res. 2022 Aug 5;6(8):e39789. doi: 10.2196/39789 (PMC9391979; doi:10.2196/39789)
Supplement: Multimedia Appendix 5 [file formative_v6i8e39789_app5.docx]

**Table S5. Post hoc testing results for the Rainbow Passage task.** t Ratios, P values and Cohen’s d effect sizes are presented for post hoc analyses of acoustic measures showing significant main effects of Time (CPP and Tilt) and Gender (f_0_). For main effects of Time, planned paired contrasts comparing scores at each time point against Day 1 (baseline) were conducted. For the main effect of Gender, Female values were compared against Male values. Statistically significant effects (P<.01) are indicated in **bold.**

| Time Points | CPP | | | Tilt | | |
| --- | --- | --- | --- | --- | --- | --- |
|  | *t* Ratio | Prob>\|*t*\| | Cohen's *d* | *t* Ratio | Prob>\|*t*\| | Cohen's *d* |
| Day 1 x Day 2 pre-session | -1.86 | .07 | 0.70 | -1.29 | .20 | 0.33 |
| Day 1 x Day 2 mid-session | -3.71 | **<.001** | 1.26 | -3.38 | **<.001** | 0.86 |
| Day 1 x Day 2 post-session | -2.35 | .02 | 1.06 | -2.22 | .03 | 0.75 |
| Day 1 x Day 3 | -0.23 | .82 | 0.27 | 0.87 | .39 | 0.33 |
| Day 1 x Day 4 | 0.07 | .94 | 0.08 | 1.28 | .20 | 0.60 |
|  | | | | | | |
| Gender | *f_0_* | | | - | | |
|  | *t* Ratio | Prob>\|*t*\| | Cohen's *d* |  |  |  |
| Female x Male | 5.83 | **<.001** | 3.07 |  |  |  |
