# Supplementary material for: Wearable Neck Surface Accelerometers for Occupational Vocal Health Monitoring: Instrument and Analysis Validation Study
Source: JMIR Form Res. 2022 Aug 5;6(8):e39789. doi: 10.2196/39789 (PMC9391979; doi:10.2196/39789)
Supplement: Multimedia Appendix 6 [file formative_v6i8e39789_app6.docx]

**Table S6. Group-based means for the Sustained Vowel task.** Means (standard deviation) for each voice metric are presented for females and males across time points. F-values, degrees of freedom, and P-values from ANOVA testing are also reported for each factor (Time, Gender) and their interaction (Time x Gender). Statistically significant effects (P<.01) are indicated in **bold.**

| Measure | Gender Group | Experimental Time Points – M (SD) | | | | | | ANOVA | | |
| --- | --- | --- | --- | --- | --- | --- | --- | --- | --- | --- |
|  |  | Day 1 | Day 2 pre-session | Day 2 mid-session | Day 2 post-session | Day 3 | Day 4 | Time | Gender | Time x Gender |
| CPP | Female | 23.91 (1.79) | 25.08 (3.38) | 26.33 (4.02) | 25.25 (2.17) | 23.58 (3.31) | 24.16 (2.97) | *F_(5,65)_*=.40  *P*=.84 | *F_(1,13)_*=.06  *P*=.81 | *F_(5,65)_*=.84  *P*=.53 |
|  | Male | 24.47 (2.23) | 24.55 (2.51) | 24.01 (4.25) | 24.40 (3.51) | 24.58 (2.03) | 24.65 (2.58) |  |  |  |
| *f_0_* | Female | 183.86 (22.17) | 190.53 (31.20) | 180.10 (32.52) | 171.94 (26.8) | 179.38 (25.92) | 184.54 (29.90) | *F_(5,65)_*=1.82  *P*=.12 | *F_(1,13)_*=25.22  ***P*<.001** | *F_(5,65)_*=.89  *P*=.49 |
|  | Male | 124.44 (22.66) | 123.97 (19.43) | 134.28 (24.02) | 114.29 (20.25) | 114.24 (17.30) | 117.63 (17.07) |  |  |  |
| H1-H2 | Female | 0.092 (0.135) | 0.188 (0.113) | 0.177 (0.153) | 0.128 (0.111) | 0.145 (0.0134) | 0.100 (0.120) | *F_(5,65)_*=2.05  *P*=.08 | *F_(1,13)_*=4.47  *P*=.05 | *F_(5,65)_*=.88  *P*=.50 |
|  | Male | 0.030 (0.061) | 0.026 (0.061) | 0.054 (0.106) | 0.016 (0.034) | 0.032 (0.049) | 0.006 (0.036) |  |  |  |
| HRF | Female | 6.40 (3.79) | 7.33 (3.53) | 7.41 (4.81) | 7.60 (4.73) | 9.06 (4.52) | 7.07 (4.45) | *F_(5,65)_*=.13  *P*=.98 | *F_(1,13)_*=.56  *P*=.47 | *F_(5,65)_*=1.06  *P*=.39 |
|  | Male | 7.11 (2.15) | 7.03 (3.60) | 7.78 (2.69) | 5.74 (0.82) | 4.66 (2.49) | 6.88 (2.58) |  |  |  |
| SE | Female | 2.19 (0.47) | 2.13 (0.51) | 2.05 (0.73) | 2.40 (0.74) | 2.27 (0.78) | 2.31 (0.56) | *F_(5,65)_=*2.43  *P*=.04 | *F_(1,13)_*=24.75  ***P*<.001** | *F_(5,65)_*=.82  *P*=.54 |
|  | Male | 3.70 (1.23) | 3.61 (1.0) | 2.90 (0.75) | 3.91 (0.49) | 3.79 (0.35) | 3.53 (0.68) |  |  |  |
| Tilt | Female | -0.079 (0.009) | -0.080 (0.011) | -0.076 (0.015) | -0.076 (0.007) | -0.083 (0.012) | -0.087 (0.014) | *F_(5,65)_*=2.62  *P*=.03 | *F_(1,13)_*=4.63  *P*=.05 | *F_(5,65)_*=1.53  *P*=.19 |
|  | Male | -0.093 (0.019) | -0.093 (0.010) | -0.075 (0.011) | -0.093 (0.013) | -0.089 (0.007) | -0.090 (0.009) |  |  |  |
| SAL | Female | 46.47 (7.00) | 52.11 (7.64) | 48.72 (9.05) | 49.14 (13.45) | 46.69 (8.53) | 49.26 (14.00) | *F_(5,65)_*=.39  *P*=.86 | *F_(1,13)_*=1.60  *P*=.23 | *F_(5,65)_*=.66  *P*=.65 |
|  | Male | 55.54 (10.24) | 51.04 (11.68) | 49.18 (5.95) | 51.77 (6.87) | 52.21 (15.53) | 57.42 (9.23) |  |  |  |
| Jitter | Female | 0.004 (0.003) | 0.005 (0.004) | 0.011 (0.012) | 0.012 (0.010) | 0.009 (0.010) | 0.010 (0.006) | *F_(5,65)_*=2.50  *P*=.04 | *F_(1,13)_*=1.00  *P*=.34 | *F_(5,65)_*=1.08  *P*=.38 |
|  | Male | 0.005 (0.005) | 0.014 (0.022) | 0.023 (0.030) | 0.015 (0.015) | 0.005 (0.004) | 0.010 (0.010) |  |  |  |
| Shimmer | Female | 0.037 (0.008) | 0.043 (0.008) | 0.055 (0.025) | 0.061 (0.022) | 0.050 (0.010) | 0.067 (0.036) | *F_(5,65)_*=3.82  ***P*=.004** | *F_(1,13)_*=5.77  *P*=.03 | *F_(5,65)_*=1.87  *P*=.11 |
|  | Male | 0.049 (0.017) | 0.061 (0.009) | 0.080 (0.041) | 0.146 (0.156) | 0.063 (0.020) | 0.071 (0.012) |  |  |  |
