# Supplementary material for: Wearable Neck Surface Accelerometers for Occupational Vocal Health Monitoring: Instrument and Analysis Validation Study
Source: JMIR Form Res. 2022 Aug 5;6(8):e39789. doi: 10.2196/39789 (PMC9391979; doi:10.2196/39789)
Supplement: Multimedia Appendix 7 [file formative_v6i8e39789_app7.docx]

**Table S7. Post hoc testing results for the Sustained Vowel task.** t Ratios, P values and Cohen’s d effect sizes are presented for post hoc analyses of acoustic measures showing significant main effects of Time (Shimmer) and Gender (f_0_ and SE). For the main effect of Time, planned paired contrasts comparing scores at each time point against Day 1 (baseline) were conducted. For main effects of Gender, Female values were compared against Male values. Statistically significant effects (P<.01) are indicated in **bold.**

| Time Points | Shimmer | | | - | | |
| --- | --- | --- | --- | --- | --- | --- |
|  | *t* Ratio | Prob>\|*t*\| | Cohen's *d* |  |  |  |
| Day 1 x Day 2 pre-session | -0.62 | .54 | 0.01 |  |  |  |
| Day 1 x Day 2 mid-session | -1.63 | .11 | 0.37 |  |  |  |
| Day 1 x Day 2 post-session | -3.97 | **<.001** | 1.18 |  |  |  |
| Day 1 x Day 3 | -0.89 | .38 | 0.05 |  |  |  |
| Day 1 x Day 4 | -1.71 | .09 | 0.31 |  |  |  |
|  | | | | | | |
| Gender | *f_0_* | | | SE | | |
|  | *t* Ratio | Prob>\|*t*\| | Cohen's *d* | *t* Ratio | Prob>\|*t*\| | Cohen's *d* |
| Female x Male | 5.02 | **<.001** | 2.50 | -4.98 | **<.001** | 1.86 |
